# In and Out of Consciousness: How Does Conscious Processing (D)evolve Over Time?

**DOI:** 10.3389/fpsyg.2017.00128

**Published:** 2017-02-02

**Authors:** Jaan Aru, Talis Bachmann

**Affiliations:** ^1^Department of Penal Law, School of Law, University of TartuTartu, Estonia; ^2^Chair of Data Science, Institute of Computer Science, University of TartuTartu, Estonia

**Keywords:** consciousness, NCC, microgenesis, time course of consciousness, transitions of consciousness

Mental information processing includes both unconscious and conscious modes and there are transitions between these two. The content of subjective experience can emerge from preconscious content, but an opposite process of loss of conscious content or its decay from subjective experience is an inevitable reality as well. Both ways of transition on the border between unconscious and conscious processing are ubiquitous. But how does this transition unfold over time? While there is quite some literature on whether conscious perception is all-or-none or graded (Sergent and Dehaene, 2004; Overgaard et al., 2006), we ask the complementary question: how does conscious perception change and evolve over time? While in phenomenological approaches in the philosophy of mind all-embracing temporal perspective has been acknowledged as crucial in understanding consciousness (e.g., anticipation, present, and retention in Husserl, 1928), in experimental paradigms only narrow temporal slices have been typically examined.

We will first focus on the knowledge about the transitions gained from studying brief visual stimuli. According to the microgenetic tradition mental content does not emerge instantaneously, in an all-or-none manner (review: Bachmann, 2000). Instead, conscious content arises as a gradual process of formation where the initial transition (there was no content and now there *is* some content) grows over to a time consuming process where subjective phenomenal content of the same intentional object matures by acquiring systematically more qualities to the preceding version of the percept. This intentional object might be a visual object, scene, memory representation etc. These microgenetically developing attributes or characteristics include subjective clarity, subjective contrast, subjective fragmentariness/exhaustiveness, coarseness/detail, subjective stability, etc. (Bachmann, 2000, 2012). In other words, conscious experience of the content pertaining to the same intentional object changes considerably over time (See also Hegdé, 2008; Breitmeyer, 2014; Pitts et al., 2014).

On the other hand (especially when brief objects typical to most of the experiments are presented) a certain experience with its phenomenal subjective content sooner or later disappears from consciousness by an analogous, but reversed gradual process—a kind of “anti-genesis” (Bachmann, 2000). Figure 1 illustrates the notion of microgenesis with its formative and disformative stages. Note that this figure is an abstraction based on the empiricial research described in Bachmann (2000). However, we hope that provocatively drawing this time course inspires researchers to investigate how it exactly looks like.

**Figure 1:**
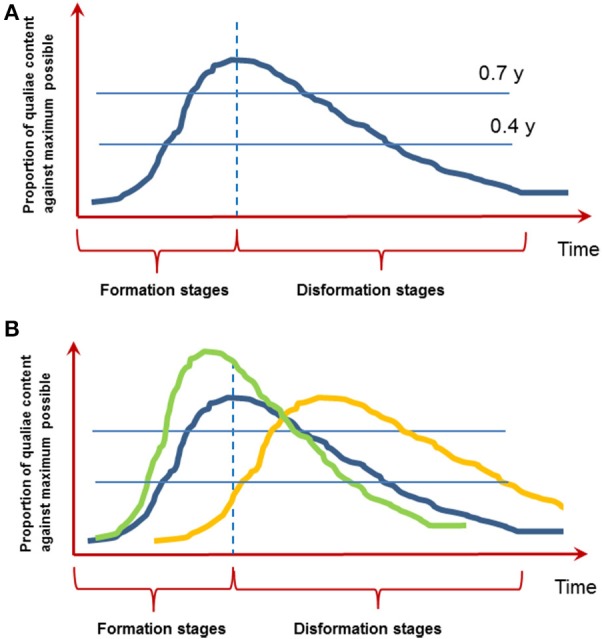
**The function of conscious experience evolving over time. (A)** Microgenesis of perception with its formative and disformative stages. The image is based on empirical research reviewed in Bachmann (2000). Note the proposed asymmetric inertia of formation and disformation. **(B)** It is conceivable that the functions over time are different for two types of conscious experience: immediate iconic perception (blue line) and slower memory-based experience (yellow line; see point 7 below). How is this shape influenced by top-down factors like attention, prior knowledge or working memory? Do these factors lead to a quicker build up and higher clarity of conscious content (green line; see point 6 below)? The units on the Y-axis are arbitrary units to be worked out.

This simple conceptualization prompts surprisingly many old and new questions.

1. What is the exact shape of this curve? How do changes in stimulus parameters (e.g., contrast, duration) change the shape?
2. Is there a kind of asymmetric inertia of formation and disformation as depicted on Figure 1? In other words, is it indeed so that disformation takes more time than formation of conscious content? To our mind, decay from consciousness seems to be slower mainly because psychophysical estimates of the speed of immediate perception by masking, temporal order estimation, speeded discrimination, and other experimental tasks are smaller than experimental estimates of the duration of immediate memory (Bachmann, 2000).
3. How to experimentally measure subjective content (a) at y_max_, (b) at any optional stage of microgenesis (e.g., y = 0.4, y = 0.7)? In fact, there are many qualities of subjective evaluation unfolding in response to stimulation or task cue (e.g., Kalmus and Bachmann, 1980; Bachmann, 1980, 2012). The most recent successfully used subjective scales include the perceptual awareness scale (PAS) capitalizing on subjective clarity ratings by subjects as well as other methods (review: Timmermans and Cleeremans, 2015).
4. A corollary question is how subjective and objective measures of consciousness relate. Do they show a mutually similar formation and disformation curve over time?
5. Is there an analogous time function for unconscious processing of content as for conscious processing? If so, how do we disentangle the conscious and unconscious processes (Miller, 2007; Bachmann, 2009; Aru et al., 2012; De Graaf et al., 2012)? Note that for example Herzog et al. (2016) propose that conscious and unconscious processing have quite different temporal characteristics, which might turn out to be helpful in disentangling them.
6. How do attention, prior knowledge or working memory content influence the shape of this curve (Figure 1B)? For example we know that all of these factors speed up the entry into consciousness and in general enhance the clarity of conscious experience (e.g., Carrasco et al., 2004; Soto et al., 2010; Aru et al., 2016). So the curve depicted on Figure 1 should rise quicker and be higher (in terms on y-units). But what about the disformation stage—do all of these factors also change how quickly the content disappears from consciousness?
7. What is the typical time course of the formative and disformative microgenetic stages? Data from ERP and MEG research on neural correlates of consciousness suggest that the initial formative stage peaks at time delays around 150–250 ms (reviewed in Bachmann, 1994; Koivisto and Revonsuo, 2010). However, P300 has also been frequently found to mark conscious experience (e.g., Sergent et al., 2005; Del Cul et al., 2007; Rutiku et al., 2015, 2016). Intriguingly, it is possible that there are two separate processes—(1) perceptual microgenesis, where conscious experience emerges fast and decays fast (possibly equal to iconic-memory decay) and (2) immediate memory-based microgenesis, where conscious experience of the same target forms a bit slower than perceptual microgenesis and decays much later than iconic delay (e.g., Sligte et al., 2008). It is even possible that while perceptual microgenetic function decays (disformation) the memory-based function is still building up (Figure 1B, yellow vs. blue line). This idea fits with the distinction between phenomenal and reflective consciousness, which are thought to depend on different types of attention (e.g., Koivisto et al., 2009).
  Now, an intriguing theoretical question appears: if one and the same stimulus-event is related to both, perceptual and immediate-memory microgenetic processes with concomitant two sets of NCC, should we then regard these NCC as different *aspects* of one NCC or a principally different, two, NCCs (Bachmann, 2015)?
8. How does the time course of subjective microgenesis relate to the time course of representational content development obtained with neural decoding and representational similarity analysis (e.g., Carlson et al., 2013; Cichy et al., 2014; Goddard et al., 2016)?
9. What are the relative roles of feedforward and re-entrant neural processes in perceptual microgenesis? For example the “reverse-hierarchy” theory of Hochstein and Ahissar (2002) suggests that global features should emerge in consciousness faster than the local features. This crucial prediction was recently confirmed (Campana et al., 2016), leading to think that conscious perception might start at the highest levels of visual processing and acquire the fine details through feedback from higher to lower levels of visual processing.
10. Are the nature and regularities of microgenesis the same when an external stimulus is becoming microgenetically formed and when a memory-image of the same stimulus is evoked and formed? More generally: are the curves of formation and disformation similar for all the transitions occurring at the threshold of consciousness? There are many examples of transitions in and out of consciousness. Can one benefit from the knowledge gathered while studying brief visual stimuli to understand processes that unfold with other types of stimuli?

To understand this last question, let us list some examples about the transition into consciousness to illustrate the heterogeneity of this kind of transformation: remembering an item or idea as cued by external instruction or question; remembering an item or idea as ignited by intrinsic associative cues; having an insight; experience of an external sensory stimulus after its initial pre-conscious processing; experience of an already presented stimulus after focusing attention on it; becoming consciously aware of an intention (agency) to act after preconscious preprocessing of the action decision; becoming consciously aware of a different aspect (feature, attribute, property, quality) of a stimulus or scene after the preceding consciously aware experience of some other aspect(s); noticing the change in a change-blindness display; noticing the target in an inattentional blindness experiment; becoming consciously aware of the Gestalt content in Mooney face or Dalmatian dog types of image after an initially “meaningless” experience; becoming aware of the words within the sine-wave speech recording; reversal of binocular-rivalry dominance in becoming aware of the suppressed stimulus; reappearance of the sensory afterimage. Is in all of these cases the emergence of conscious content gradually evolving over time?

There are also many examples for the transition out of consciousness: fading of the conscious percept; fading of iconic memory; loss of thought or imagery content; loss of explicitly experienced WM content; loss of conscious awareness of a stimulus after refocusing attention elsewhere; loss of a certain aspect (feature, attribute, quality) of conscious perception of a discontinued stimulation while other aspect(s) sustain; loss of conscious perception of a binocular-rivalry stimulus when it becomes suppressed; fading of perceptual content (e.g., color, spatial contrast modulation, luminance contrast step gradient) due to sensory adaptation; fading of afterimage. Is the fading of conscious content gradually evolving over time in all of these cases?

Gathering such a list leads yet again to interesting questions. For example, which transitions are *reversible* and which ones not? Loss of formed Gestalt content back to meaningless array of elements seems difficult; re-establishment of change-blindness/inattentional blindness seems impossible; after hearing the words within the sine-wave speech it is impossible to go back to hearing noise. What could this small set of non-reversibility tell us about the neural mechanisms of consciousness? The list of these phenomena seems to suggest that conscious experience is heavily influenced by prior knowledge—once insightful knowledge about a particular stimulus is established, it is hard or even impossible to remove it.

More importantly, the variety of examples leads to the question whether there are general mechanisms and regularities underlying all of these phenomena. Do all these other types of transitions share some of the key features with the transitions happening in visual perception (Figure 1)? In visual perception it is relatively straightforward to “slice up” perception with techniques like visual masking (Bachmann, 1994; Bachmann and Francis, 2013), but even then studying the time course of visual perception is a time consuming and a difficult endeavor (Bachmann, 2000). Is it possible or even meaningful to try to do it with other types of transitions? How would one proceed with “slicing up” memory retrieval, Gestalt perception or insight formation? We do not have definitive experimental approaches, but we consider these questions to be important to put forth and to explore.

The present manuscript had a few aims: (1) we wanted to emphasize that conscious content evolves and changes over time, (2) we noted that the exact time course of how conscious content evolves over time is yet unknown and tentatively drew a time course to provoke more research in this direction, (3) we wanted to demonstrate that thinking about the time course of conscious processing prompts many interesting and intriguing questions, (4) finally we asked how general are such microgenetic regularities—do all kinds of transitions in and out of consciousness have gradual formation and disformation (Figure 1)? On the way we also seem to have stumbled on a few novel concepts applicable in studying dynamics of conscious experience—the formation/disformation *(a)symmetry, reversibility*, and the possibility of having two different NCCs for the same perceived object. We hope that some of these ideas and concepts are beneficial for unraveling the neural mechanisms of consciousness.

## Author contributions

TB conceived the initial ideas, JA expanded them, both JA and TB discussed the ideas and contributed to writing the manuscript

### Conflict of interest statement

The authors declare that the research was conducted in the absence of any commercial or financial relationships that could be construed as a potential conflict of interest.
